# Supporting the Sensory Panel to Grade Virgin Olive Oils: An In-House-Validated Screening Tool by Volatile Fingerprinting and Chemometrics

**DOI:** 10.3390/foods9101509

**Published:** 2020-10-21

**Authors:** Beatriz Quintanilla-Casas, Marco Marin, Francesc Guardiola, Diego Luis García-González, Sara Barbieri, Alessandra Bendini, Tullia Gallina Toschi, Stefania Vichi, Alba Tres

**Affiliations:** 1 Departament de Nutrició, Ciències de l’Alimentació i Gastronomia, Campus de l’Alimentació de Torribera, Facultat de Farmacia i Ciències de l’Alimentació, Universitat de Barcelona, 08921 Santa Coloma de Gramenet, Spain; beatrizquintanilla@ub.edu (B.Q.-C.); marin.marco95@gmail.com (M.M.); fguardiola@ub.edu (F.G.); atres@ub.edu (A.T.); 2 Institut de Recerca en Nutrició i Seguretat Alimentària (INSA-UB), Universitat de Barcelona (UB), 08921 Santa Coloma de Gramenet, Spain; 3 Instituto de la Grasa (CSIC), 41013 Sevilla, Spain; dlgarcia@ig.csic.es; 4 Department of Agricultural and Food Science, Alma Mater Studiorum-Università di Bologna, 47521 Cesena, Italy; sara.barbieri@unibo.it (S.B.); alessandra.bendini@unibo.it (A.B.); tullia.gallinatoschi@unibo.it (T.G.T.)

**Keywords:** virgin olive oil, sensory quality, volatile compounds, HS-SPME–GC–MS, chemometrics, panel test, validation, untargeted

## Abstract

The commercial category of virgin olive oil is currently assigned on the basis of chemical-physical and sensory parameters following official methods. Considering the limited number of samples that can be analysed daily by a sensory panel, an instrumental screening tool could be supportive by reducing the assessors’ workload and improving their performance. The present work aims to in-house validate a screening strategy consisting of two sequential binary partial least squares-discriminant analysis (PLS-DA) models that was suggested to be successful in a proof-of-concept study. This approach is based on the volatile fraction fingerprint obtained by HS-SPME–GC–MS from more than 300 virgin olive oils from two crop seasons graded by six different sensory panels into extra virgin, virgin or lampante categories. Uncertainty ranges were set for the binary classification models according to sensitivity and specificity by means of receiver operating characteristics (ROC) curves, aiming to identify boundary samples. Thereby, performing the screening approach, only the virgin olive oils classified as uncertain (23.3%) would be assessed by a sensory panel, while the rest would be directly classified into a given commercial category (78.9% of correct classification). The sensory panel’s workload would be reduced to less than one-third of the samples. A highly reliable classification of samples would be achieved (84.0%) by combining the proposed screening tool with the reference method (panel test) for the assessment of uncertain samples.

## 1. Introduction

As stated in the International Olive Council (IOC) standard [1], virgin olive oils are obtained from the fruit of the olive tree (*Olea europaea* L.) solely by mechanical or other physical means, under conditions that do not alter the oil. Virgin olive oil trading implies a previous compulsory assessment of its quality by chemical-physical analyses and organoleptic evaluation [2], leading to grade this food product into three commercial categories: extra virgin olive oil (EVOO), virgin olive oil (VOO) and lampante–non-edible olive oil (LOO). The current official method for the organoleptic assessment is the evaluation by the panel test [3]. This evaluation is important to get a global picture of the sensory properties of virgin olive oil and it is particularly precious and hardly replaceable when describing the sensory profile of EVOO under quality schemes, such as Protected Designation of Origin [4]. Although the panel test has been responsible for improving the quality of virgin olive oils in the last years [5], many authors agree on the controversies about this method [4,5,6]. As a matter of fact, since it was first introduced into European Regulations in 1991, it has undergone many revisions as the result of a continuous study of its performance [7]. Even so, the sensory panel test still presents some drawbacks linked to the method’s nature, such as the limited number of samples assessed per day and misalignment between panels, among others [5]. It is also important to remember that the global production of virgin olive oil is growing at worldwide scale, while the number of recognized sensory panels is limited [6]. For all these reasons, setting up an instrumental screening tool that supports the sensory panel has become a need that should be fulfilled. In the last years, many works intended to achieve this purpose through different ways. Generally, volatile organic compounds (VOC) are the most pursued markers, due to their widely reported relationship with the sensory properties of virgin olive oil [8,9,10]. Their analysis has been performed by different methods, such as (i) gas chromatography–mass spectrometry (GC–MS) [11,12,13,14], (ii) gas chromatography–ion mobility spectrometry (GC–IMS) [15,16,17,18,19], (iii) proton transfer reaction–time of flight-mass spectrometry (PTR–ToF-MS) [4,20] and (iv) MS without a previous separation step [21,22,23,24]. Rapid methods like flash gas chromatography (FGC) electronic nose have also been used for this purpose [25,26]. In some cases, non-volatile compounds also related with sensory perception have been used to develop classification methods [22,23,27,28]. All these analytical approaches have been combined with different chemometric tools to develop classification strategies, from basic ones to the most advanced ones such as deep learning [19] or data fusion techniques [22]. However, if the goal is to provide a fit-for-purpose tool that could eventually be applied in many laboratories to support the panel test, it is necessary to combine a suitable analytical method with a chemometric approach that is as simple as possible. In this sense, in a preliminary work [14], we presented a proof of concept for an instrumental screening tool based on virgin olive oil volatile fingerprinting by head space-solid phase micro extraction (HS-SPME) and GC–MS analysis. This analytical method is known to be simple, relatively fast, automatable, affordable and highly sensitive [13,29,30,31]. Besides, that analytical tool is based on the use of raw chromatographic data, also called fingerprint, an approach that allows working with the full analytical information of samples, increasing the amount of information used in modelling as well as overcoming the identification and integration troubles of the conventional target approach [32]. In that work [14], a hierarchical model based on two binary partial least squares-discriminant analysis (PLS-DA) models was proposed as a classification strategy. Even though the decision criterion in binary discriminant models is usually a single classification threshold, in that case an uncertainty range was set to identify boundary samples. In this way, the instrumental screening tool allowed classifying clear samples into EVOO, VOO or LOO and left the boundary ones that fall into the uncertain range to be further assessed and graded by the sensory panel, thus reducing its workload [14].

These satisfactory results prompted us to keep improving the tool to achieve a reliable instrumental screening tool as well as to bring its real future application one step closer. For this, it was still necessary to (i) improve the sample database, increasing sampling representativeness, (ii) set the terms for the validation of the analytical fingerprint method, and (iii) optimize and validate the classification strategy with the updated sample database. All these steps are addressed in this article as they need to be accomplished before the tool can be submitted to a system challenge (applying it to data obtained from different GC–MS instruments and labs) and eventually to an interlaboratory study.

Regarding sampling, an increase in the number of samples, including samples from at least two crop years, aiming to add natural variability to the classification model, is broadly recommended [15].

A fingerprinting method needs to undergo a validation process in order to be transferred to routine analysis. The first step prior to interlaboratory validations or peer validation studies consists of an in-house validation. First, this validation shall address the analytical fingerprint obtained by SPME–GC–MS to verify its repeatability and reproducibility and, subsequently, shall focus on the classification model. In target-based methods, validation procedures for both analytical methods and statistical models are well defined [33,34], given that results are usually evaluated compound by compound using univariate statistics. In contrast, since data obtained by fingerprinting approaches are assessed on a pattern level using multivariate models [35], conventional performance criteria adopted for the former methods are not applicable as such to the latter. Specific guidelines are necessary for this purpose, but they are still lacking. Some authors proposed method validation strategies for non-targeted metabolomic analysis [36,37], but they could adapt the target validation procedure because they sought the validation of biomarkers where target-like steps such as analyte quantification are carried out in the end. In contrast, the fingerprinting approach followed in the present study is not intended to carry out identification and quantification of single compounds. Indeed, as defined by Riedl et al. [35], the “spectral or chromatographic fingerprint” already represents the “analyte”. Given the lack of clear-cut guidelines for the analytical validation of fingerprinting methods, we proposed and evaluated a procedure for the in-house assessment of the repeatability of the analytical signal, which is one of the constraints when transferring fingerprinting methods. 

Regarding the optimization and validation of the classification strategy, to maximize its performance, the models’ uncertainty ranges were set by means of receiver operating characteristics (ROC) analysis [38] in which high sensitivity and specificity can be ensured. Moreover, both internal and external validation were performed in seven independent runs. This external validation also included the validation of the classification criteria based on the uncertainty ranges from ROC analysis, to assess up to which extent they were influenced by the samples in the training set and how the uncertainty ranges performed in classifying new samples. 

Therefore, the present work aimed to in-house validate the analytical fingerprint, to evaluate the suitability of ROC curves to set the uncertainty ranges and to validate the screening tool—built with a high number of samples from two crop years and graded by six different sensory panels—to support virgin olive oil sensory panels.

## 2. Materials and Methods 

### 2.1. Olive Oil Samples

A total of 305 virgin olive oil samples, from two consecutive crop years (2016–2017 and 2017–2018) produced in several EU and non-EU countries, were sensory-assessed and graded as EVOO, VOO or LOO by six official sensory panels according to the International Olive Council procedure [3]. The panels were located in different countries (Germany, France, Croatia, Italy, Turkey, Slovenia), and all of them were partners in the OLEUM project (“Advanced solutions for assuring authenticity and quality of olive oil at a global scale”, funded by the European Commission within the Horizon 2020 Programme 2014–2020, GA no. 635690). The samples included in the model (*n* = 305) were those for which there was a consensus on their category among the six panels, both if the consensus was reached at first or after a formative reassessment of the panels to correct misalignments [5]. Thus, the sample set finally comprised 122 EVOO, 108 VOO and 75 LOO. This sample set included VOO and LOO samples whose perceived main defect was rancid, musty-humid-earthy or fusty-muddy. Comprehensive information about the samples is available in the Supplementary Material (Table S1). The samples were kept at −18 °C until analysis.

### 2.2. Chemicals and Reagents

Hexanal (CAS Number 66-25-1; assay 98%), 1-hexanol (CAS Number 111-27-3; assay ≥ 99.9% (GC)), (*Z*)-3-hexenyl acetate (CAS Number 3681-71-8; assay ≥ 98.0%), (*E*)-2-heptenal (CAS Number 18829-55-5; assay ≥ 95%), pentanoic acid (CAS Number 109-52-4; assay ≥ 99.8%), were all from Sigma-Aldrich (St. Louis, MO, USA).

### 2.3. Head Space-Solid Phase Microextraction (HS-SPME)

The extraction of VOC from virgin olive oil samples was performed according to Vichi et al. (2003) by means of a Combi-pal autosampler (CTC Analytics, Zwingen, Switzerland) configured for HS-SPME. First, 2 g of olive oil sample was placed into a 10 mL glass vial fitted with a polytetrafluoroethylene (PTFE)/silicone septum (Scharlab, Barcelona, Spain)). Then, the vial was kept at 40 °C under agitation for 10 min, for sample conditioning. After that, a divinylbenzene/carboxen/polymethylsiloxane (DVB/CAR/PDMS) fibre (2 cm length, 50/30 thickness) from Supelco (Bellefonte, PA, USA) was exposed for 30 min to the sample headspace to extract VOC. Finally, the analytes were desorbed by placing the fibre in the gas chromatograph injector port (260 °C) with an SPME injector sleeve (0.75 mm ID) for 10 min, maintaining it for the first 5 min in split-less mode.

### 2.4. Gas Chromatography–Mass Spectrometry (GC–MS)

#### 2.4.1. Instruments and Procedures

The separation of VOC was performed by an Agilent Technologies 6890N Network GC system (Agilent Technologies, Palo Alto, CA, USA) equipped with a Supelcowax-10 column of 60 m × 0.25 mm i.d., 0.25 μm film thickness (Supelco, Bellefonte, PA, USA). The GC oven temperature was held at 40 °C for the first 10 min, then increased to 150 °C at 3 °C/min and finally to 200 °C at 15 °C/min, holding it at that ending temperature for 5 min. The carrier gas was helium, with a flow rate of 1.5 mL/min. The MS was an Agilent Technologies 5975C inert XL quadrupolar analyser; ion source and transfer line temperature were 230 °C and 275 °C, respectively. The MS analyser worked in full scan mode (*m/z* range from 30 to 300), 5.1 scans/s and electron energy set to 70 eV.

#### 2.4.2. Analytical System Suitability

To assess the analytical system suitability prior to the in-house validation and the analysis of the samples, we set basic performance criteria to establish some minimum requirements in terms of sensitivity and chromatographic resolution.

Analytical sensitivity, defined here as the quotient of the change in an indication of a measuring system and the corresponding change in a value of a quantity being measured, was checked by analysing a standard solution prepared by spiking 2 g of refined olive oil with hexanal (0.05 mg/kg), 1-hexanol (0.05 mg/kg) and pentanoic acid (0.5 mg/kg). These compounds were selected because they belong to different chemical families (aldehydes, alcohols and organic acids) and are present along the chromatogram. Here, the signal-to-noise ratio for each compound should be >3 at these conditions. This was performed in duplicate.

Minimum chromatographic resolution (*R*), defined as the difference between peak retention times divided by the average peak width, was evaluated by analysing 2 g of a standard solution of (*Z*)-3-hexenyl acetate and (*E*)-2-heptenal at the concentration of 5 mg/kg in refined olive oil. These compounds were selected to calculate R because they elute at close retention times. At experimental *R* < 1, the chromatographic conditions must be optimized.

### 2.5. In-House Validation of the Analytical Outcome

First, a pooled quality control (QC) sample was prepared by mixing equal volumes of six representative control virgin olive oil samples: two EVOO and four LOO. The latter included the following sensory defects (expressed as median of the intensity; *M*): rancid (*n* = 2, *M* > 4) (LR), fusty-muddy sediment (*n* = 1, *M* > 5) (LF) and fusty-muddy sediment + musty-humid-earthy (*n* = 1, *M* > 5 each) (LFH). Then, seven replicates of this pooled QC were analysed by the same operator with the same equipment, working with the same instrument operative conditions: (i) within the same day to assess intra-day repeatability and (ii) in different days to measure inter-day repeatability. The control samples were also analysed in duplicate.

The total ion chromatograms (TIC) were extracted from minute 5, including the whole *m/z* range acquired. All TIC were aligned by the Icoshift algorithm [39] in Matlab R2018b^®^ (The MathWorks Inc., Natick, MA, USA) to correct the retention time shift between samples. 

The relative standard deviation percentage (RSD %) was calculated for each data point in the aligned chromatogram to assess intra- and inter-day repeatability [40], excluding those scans whose intensity was below the mean noise plus three folds the standard deviation. The mean noise was assessed within a representative range of more than 300 consecutive scans of the TIC without volatile compound peaks.

The precision of the analytical outcome was also evaluated through a principal component analysis (PCA) (*n* = 26) performed with the aligned volatile fingerprint of the QC (intra-day and inter-day replicates) and the control virgin olive oil samples that conformed the QC. Precision was evaluated by means of the score scatter and residual standard deviation plots [41]. The latter plot gives a distance to the model measurement, so samples below the critical value are judged as non-deviating. Here, the normalized critical value was set at 0.05 according to a significance level of 95%.

### 2.6. Chemometrics

Raw data were obtained as explained in Section 2.5. Due to the high number of samples (*n* = 305) and data complexity, the Icoshift algorithm was applied to five individual analytical batches, in order to pre-align them before aligning them altogether. The resulting aligned data matrix, consisting of as many columns as variables (16,347 data points) and as many rows as samples (*n* = 305), was imported into SIMCA software v13.0© (Umetrics Sartorius-Stedim, Malmö, Sweden). 

**Figure 1:**
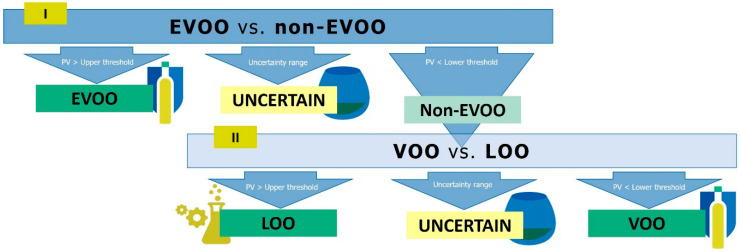
Flow chart of the two-step hierarchical classification strategy to classify virgin olive oils into their quality grades: extra-virgin (EVOO), virgin (VOO) and lampante (LOO) olive oils. PV: predicted values.

After data pre-processing by scaling to unit variance and mean centring, a PCA with all samples (*n* = 305) was performed, aiming at multivariate data exploration and detection of potential outliers through Hotelling’s *T*^2^ and *Q*-residuals. Then, PLS-DA was applied to develop a 2-step hierarchical classification strategy with the full sample set (*n* = 301), outliers excluded. Figure 1 summarizes the hierarchical classification strategy.

PLS-DA is a supervised discriminant technique that finds the most different features between categories while minimizing those variables not related to a given category; it is based on finding the maximum correlation between the data and each of the categories, being able to handle noise in the data. In this approach, two consecutive binary PLS-DA models discriminated among the three commercial categories of virgin olive oil in two steps, as our previous work showed that this was more successful than a PLS-DA model with the three categories [14]. First, the PLS-DA model EVOO vs. non-EVOO samples (values of the PLS dummy variable were 1 for EVOO and 0 for non-EVOO) was used to discriminate these classes by means of predicted values (PV). Then, a second PLS-DA model, VOO vs. LOO (values of the PLS dummy variable were 1 for LOO and 0 for VOO), was applied to predict the category of non-EVOO samples. Both PLS-DA models were optimized by leave 10%-out cross-validation. This hierarchical classification strategy was also followed by subsequent studies in this matter [18,26]. Then, as suggested by Quintanilla-Casas et al. [14], the classification criterion included an uncertainty range to which boundary samples were assigned, those with PV within the uncertainty range. Here, ROC analysis was applied to both PLS-DA models in order to set the thresholds of the uncertainty ranges. Therefore, two ROC curves (one for EVOO vs. non-EVOO models and another one for VOO vs. LOO models) were built up with the PV obtained in the leave 10%-out cross-validation and the true category for each sample in each binary model. A ROC curve plots the sensitivity and 1-specificity values that are obtained when the PV threshold to assign samples to a diagnostic category varies (in this case, the EVOO category in the EVOO vs. non-EVOO model, and the LOO category in the VOO vs. LOO model) [36]. In order to maximize the classification performance, the PV that led to sensitivity (true positive rate) and specificity (true negative rate) equal to 1 in each PLS-DA were respectively taken as the lower and the upper thresholds of the uncertainty range (Figure 1). Thus, in the first model, virgin olive oil samples with PV above the fixed upper threshold were classified as EVOO, those with PV below the lower threshold (non-EVOO) were subjected to further classification by the second model and those with PV in between were considered uncertain to classify. On the other hand, the second model classified the non-EVOO as LOO (PV > upper threshold), VOO (PV < lower threshold), or uncertain (PV between thresholds) (Figure 1). 

The sample set was randomly split seven times, each time into a training set, with the 80% of samples from each category (*n* = 241; 98 EVOO, 86 VOO and 57 LOO), and an external validation set with the remaining 20% of samples (*n* = 60; 24 EVOO, 22 VOO and 14 LOO). The 2-step hierarchical classification strategy was developed and cross-validated (leave 10% out) with each of the seven training sets. In each PLS-DA, the number of latent variables (LVs) was selected according to the lowest root-mean-square error of cross-validation (RMSEcv) value and the highest prediction power (Q^2^). Undesirable features, such as model overfitting and random behaviour, were checked by permutation tests (*n* = 20 permutations) and analysis of variance (ANOVA) on the cross-validation residuals, respectively. ROC analysis was applied to PV values from each individual PLS-DA in order to set its own uncertainty range. Therefore, 14 ROC curves (7 random training sets per 2 PLS-DA models) were built up. 

Then, each of the 2-step hierarchical classifications of the seven training sets was externally validated by using it to predict the category of the samples in the corresponding validation set (*n* = 60), using as classification criteria the uncertainty ranges obtained from the corresponding ROC curves. Thus, in each of the seven sets, both the PLS-DA models and the uncertainty ranges used as classification criteria were externally validated by using them on new samples not used to develop the model nor to set the classification thresholds. Mean (%) and standard deviation of correctly classified and uncertain samples were calculated from the seven models’ results, for each commercial category (EVOO, VOO and LOO). 

## 3. Results and Discussion

### 3.1. In-House Validation of the Analytical Outcome

Although there is not a consensus yet on the procedure to validate fingerprinting analytical methods, and precise guidelines have not been established, ensuring the quality and reproducibility of analytical results is a necessary condition to posit a method as an authentication screening tool. As fingerprinting methods are not aimed to the identification and quantification of analytes but to find distinctive patterns specific for a given food category (i.e., virgin olive oil commercial category) in raw analytical signals (i.e., chromatograms), the main constraint is providing a repeatable and reproducible signal with enough sensitivity to collect valuable information from the samples. For this purpose, we proposed some performance criteria to evaluate the repeatability of the analytical outcome, provided that the system suitability fulfilled the minimum requirements. 

On the one hand, as suggested by Allwood et al. [40], we evaluated the RSD of each data point in the chromatogram that had an intensity above mean noise. After noise subtraction, the number of variables decreased to around 70% of the initial signals. The FDA indicates that RSD values of 15% are generally acceptable as regards analytical variability for target analysis, except for concentrations close to the detection limit where an RSD of 20% is acceptable [34]. Even though in this work we chose a fingerprinting approach, where more variation is expected, the previous reference was used as a benchmark towards the repeatability assessment. Here, 99.3% and 98.0% of the variables presented RSD equal or below 15% when assessing intra-day (Supplementary Material, Figure S1a) and inter-day (Supplementary Material, Figure S1b) repeatability, respectively, which was in accordance with the FDA recommendation. The RSD was also used to assess chromatographic fingerprints obtained by FGC, achieving comparable outcomes [26]. When the RSD was plotted versus the signal intensity for intra-day (Supplementary Material, Figure S1a) and inter-day repeatability (Supplementary Material, Figure S1b), both plots showed that the higher the signal intensity, the lower the RSD. This agreed with the trend described by the Horwitz’s equation, formulated for targeted methods, and proved that the repeatability was strongly correlated with the intensity of the variables [42]. 

**Figure 2:**
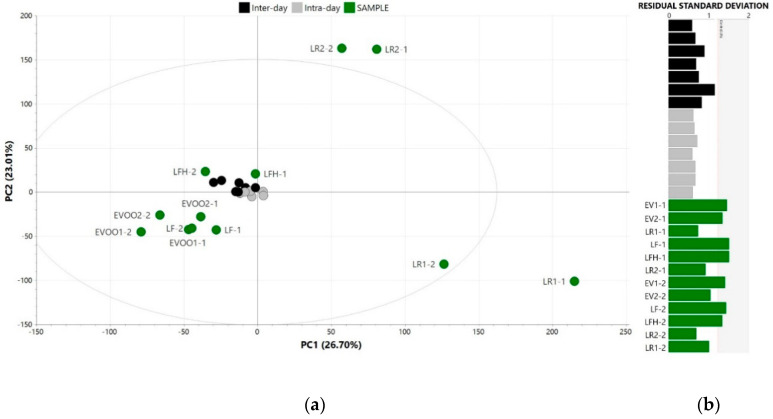
Principal component analysis (scaled to unit variance and mean centred) of pooled quality control samples analysed for repeatability (intra- and inter-day) assessment along with the single control samples: (**a**) Score scatter plot and (**b**) residual standard deviation plot. LR: lampante olive oil with rancid defect; LF: lampante olive oil with fusty-muddy defect; LFH: lampante olive oil with fusty-muddy and musty-humid-earthy defect.

On the other hand, the precision of the analytical signal was also evaluated by the clustering of QC samples in a PCA (four principal components, 73.3% of explained variance), as proposed by Dudzik et al. [41] for untargeted metabolomics, which supported the above-mentioned findings. Here, tight clusters at the centre of the score scatter plot were observed for the QC samples analysed for intra-day and inter-day repeatability, which according to Dudzik et al. [41], indicated a precise analytical outcome (Figure 2a). These QC clusters were located close to EVOO and LFH control samples, pointing out the strong weight of the variables related to these sensory profiles (Figure 2a). Furthermore, the residual standard deviation plot of the PCA showed that both intra-day and inter-day replicates were under the critical value (0.05), confirming no deviations in their distance to the PCA model (Figure 2b).

### 3.2. Raw Data Alignment, Pre-Processing and Exploration

First, a global alignment of the whole sample set was carried out, but since each analytical batch consisted of a reduced number of samples with smaller retention time shifting among them, the pre-alignment per batch was more straightforward and more convenient in terms of computational time. Given the difficulty of finding a parameter to compare the two alignment approaches, we compared the results from PCA and PLS-DA obtained with the data of the full sample set aligned by both approaches. Even though the results were highly similar, which is a good indicator of the weak influence exerted by the alignment approach on the model, the “per batch” approach was slightly more efficient in terms of explained variance in PCA and in better classification results of cross-validation with less LVs in PLS-DA (Supplementary Material, Table S2).

Among the pre-processing techniques applied to the volatile fingerprinting data, unit variance scaling and mean centring offered the highest model’s performance. Through PCA (four principal components, 39.4% of explained variance), four LOO samples were identified as outliers according to Hotelling’s *T*^2^ and *Q*-residuals parameters, leaving a sample set of 301 virgin olive oils to apply the classification models.

### 3.3. Development of the Hierarchical Classification Strategy

Following the strategy summarized in Figure 1, the classification approach consisted of two consecutive binary PLS-DAs: the first model discriminated EVOO from non-EVOO samples, while the second one classified the non-EVOO samples into VOO and LOO. As discussed in our previous work [14], setting an uncertainty range for each binary discriminant model resulted more appropriate than the establishment of a single threshold to classify the samples, since the purpose was to develop a screening tool to reduce panels’ assessment workload. In this way, virgin olive oil samples outside these ranges would be directly assigned to a given category, whereas the sensory panel would only determine the commercial category of uncertain samples (Figure 1). In the present study, the uncertainty range was not set according to conventional values, as it was done in our previous work [14]; instead, the range thresholds were obtained from each classification models’ performance through ROC curves. This allowed us to propose specific ranges for the models, maximizing the accuracy of the instrumental screening tool [43], given that thresholds were set at PV where sensitivity and specificity were maximum (equal to 1). Therefore, a full model (*n* = 301) was developed and cross-validated; ROC analysis was performed for both PLS-DA conforming the classification strategy to obtain the uncertainty ranges (Table 1). In order to externally validate the classification approach, the sample set was split into (a) a training set with 80% of the samples from each class (*n* = 241); (b) a validation set with the remaining 20% (*n* = 60). The hierarchical classification was then developed seven times with the training sets: models and classification criteria based on ROC results were established. Table 1 shows the thresholds of the uncertainty ranges for each of the seven models based on the ROC curves and the percentage of samples that fell into them. 

The mean of uncertain samples was 13.8% and 2.5% for EVOO/non-EVOO and VOO/LOO models, respectively. The remaining samples in both models were all correctly classified into one of the given categories in cross-validation, since we had set the models’ boundaries at their best classification performance (sensitivity and specificity equal to 1).

In the EVOO/non-EVOO model, the lower thresholds were more similar to each other compared to the upper thresholds, reflecting that sensitivity was less variable than specificity. Only two out of seven VOO/LOO models presented an uncertainty range, given that the accuracy in most of the cases was 100%. 

### 3.4. External Validation of the Instrumental Screening Tool

The models developed with the seven sets were used to classify the samples of the corresponding seven validation sets, using as classification criteria their respective uncertainty ranges. The results of the external validation of the proposed instrumental screening tool are collected in Table 2.

Among the classified samples, a mean of 78.9% of the samples graded only by the instrumental screening tool were correctly assigned, being the EVOO category the one with the best performance. Regarding the uncertainty rate, a mean of 23.3% of the virgin olive oil samples were identified as boundary and would need the panel test assessment, meaning that the workload of the sensory panel would have been reduced to almost one-third of the total sampling. Here, we found differences among the categories; the LOO presented the fewest uncertain samples. This fact could be explained by the lack of fruity sensory attributes and the high presence of several defects, features that allow a clearer classification. Finally, bearing in mind that currently the sole and only reference method to sensory grade virgin olive oils is the panel test, a full correct classification is assumed from this evaluation. Therefore, considering that the uncertain samples would be assessed by the sensory panel and would be correctly graded, we can state that a mean reliable assignment of 84% would be reached by combining the instrumental screening tool and the panel evaluation, with a panel workload reduction to one-third of the total sampling. Therefore, the classification rate obtained by the present strategy is comparable to those obtained by other approaches [11,12,13,15,16,21], but it is based on a simple, relatively fast, automatable and widespread technique. 

As it has been mentioned above, misalignments can sometimes occur between different panels. Here, a group of 24 VOO and LOO samples were reported as boundary by the sensory panels’ coordinator because initially there was a lack of agreement among the panels and they needed a formative reassessment after which a consensus was reached [5]. In other studies, olive oils are submitted to a single panel that determines their category. Since this category is taken as definite, any error or misalignment would affect the identity of the samples and model development and validation. However, we were able to detect these boundary samples thanks to the joint work of the six sensory panels, which allowed us to obtain more reliable results, as we could be sure that the category of the samples had been correctly assigned by the panels. To understand how these boundary samples influenced the classification efficiency of the instrumental screening method, we examined the identity of the misclassified and uncertain samples during model external validation. We observed that several of the VOO and LOO that were misclassified or identified as uncertain in any of the sets of the validation step pertained to this boundary group: main perceived defect (MPD) fusty-musty: samples 187, 190, 285 and 290; MPD musty-humid-earthy: samples 286 and 289; MPD rancid: samples 189, 192, 193, 287, 288 and 301, Supplementary Material (Table S1). Interestingly, 12 out of those 24 boundary oils were correctly classified by the instrumental method according to the reassessment panel’s results. This fact highlights the great support that the instrumental screening tool would provide to the sensory panel also in case of boundary samples. 

Moreover, to ascertain the role of the type of sensory defect in classification efficiency, we investigated whether the successfulness of the classification depended on the presence of a given defect in a sample. Table 3 shows the results of the external validation of VOO and LOO samples according to their MPD (rancid, fusty-muddy or musty-humid-earthy). According to the global results of Table 3, the highest uncertainty rate was found for VOO and LOO samples whose MPD was rancid, which also had a lower classification rate. This may be because oxidation VOC are present in virgin olive oils of all categories, even in different concentrations. Samples with fusty-muddy and, in particular, musty-humid-earthy features as MPD generally performed better in terms of percent of uncertain samples and classification as non-EVOO, although we cannot make a clear statement about the classification into VOO and LOO classes of the latter due to the limited number of samples with this MPD. We can conclude that, although the classification efficiency depends on the type of MPD present in virgin olive oils, the correct classification obtained for samples presenting one of the three evaluated MPD was satisfactory.

## 4. Conclusions

Due to the relevance of sensory aspects to discriminate commercial categories of virgin olive oil, an organoleptic assessment to grade oils as EVOO, VOO or LOO (not edible without refining) is necessary before placing them on sale. A limited number of recognized panels are available for the increasing worldwide virgin olive oil production, thereby the panel’s work becomes overwhelming. This, along with the limited number of samples that can be assessed per day, puts forward the need to dispose of an instrumental screening tool supporting the sensory panel by reducing its workload and allowing the improvement of its performance. 

With that objective, the present work proposes a “fit-for-purpose” tool based on the volatile fingerprint of virgin olive oils obtained by HS-SPME–GC–MS. This screening tool was developed using more than 300 virgin olive oil samples, from many different cultivars as well as geographical origins, graded as EVOO, VOO and LOO by six recognized sensory panels. Here, an in-house validation of the analytical method for fingerprinting approaches was proposed and performed, providing promising outcomes. Also, an external validation of the hierarchical classification strategy, based on two binary PLS-DA, was carried out, allowing excellent classification results. Hence, the joint application of the instrumental screening tool with the current reference method would result in a highly reliable classification of virgin olive oil samples, with an error rate under 15%; also, the performance of the panel test would improve since its workload would be reduced below the 30% of the total sampling.

Nevertheless, a big effort is still necessary to validate chromatographic fingerprinting methods. According to Riedl [33], a validation scheme for multivariate models dealing with fingerprint data ends with a system challenge, which comprises system conditions or sample characteristics different from those under which the model was developed and thus involves the use of different GC–MS instruments, columns, laboratories, etc. A system challenge would allow establishing whether samples analysed by different laboratories can be classified with the same model or whether it is necessary to develop a database and a tailor-made model for each laboratory. This last step is essential to understand if this fingerprinting method could be introduced as a new routine method in many labs or even become an official method.

## Figures and Tables

**Table 1:** Uncertainty ranges obtained from receiver operating characteristics (ROC) curves and uncertain samples for each classification model, obtained during internal validation of seven different subsets (leave-10%-out cross-validation).

	1st PLS-DA (EVOO vs. Non-EVOO) 7 LVs; *Q*^2^ >0.4; RMSEcv < 0.37; ANOVA *p*-Value < 0.05			2nd PLS-DA (VOO vs. LOO) 5–7 LVs; *Q*^2^ >0.4; RMSEcv < 0.33; ANOVA *p*-Value < 0.05		
Set	Lower Threshold ^1^	Upper Threshold ^2^	Uncertain Samples ^3^ (%)	Lower Threshold ^1^	Upper Threshold ^2^	Uncertain Samples ^3^ (%)
**Full model**	0.354	0.752	15 (49/301)	0.434	-	
**Subsets**						
Set 1	0.397	0.503	4.2 (10/241)	0.473	-	
Set 2	0.427	0.836	17.4 (42/241)	0.418	0.514	2.8 (4/143)
Set 3	0.383	0.771	15.4 (37/241)	0.468	-	
Set 4	0.310	0.497	6.6 (16/241)	0.442	-	
Set 5	0.370	0.748	14.5 (35/241)	0.491	-	
Set 6	0.426	0.812	16.6 (40/241)	0.483	-	
Set 7	0.411	0.760	22.2 (27/241)	0.425	0.502	2.1 (3/143)
**Mean**	0.389	0.704	13.8	0.457	0.482	2.5
**SD**	0.041	0.142	6.31	0.029	0.024	0.5

PLS-DA: partial least squares-discriminant analysis, LV: latent variable, *Q*^2^: prediction power of the model, RMSEcv: root-mean-square error of cross-validation, SD: standard deviation. ^1^ Models’ sensitivity was equal to 1 for predicted values under this threshold; ^2^ Models’ specificity was equal to 1 for predicted values above this threshold; ^3^ The numbers between parentheses correspond to the number of uncertain samples/number of samples in the training set.

**Table 2:** External validation results of the instrumental screening tool (classified correctly and uncertain samples for EVOO (*n* = 24), VOO (*n* = 22) and LOO (*n* = 14) samples) and the final reliable assignment considering the screening tool’s plus the panel test’s classification results.

Instrumental Screening Tool							Screening Tool + Reference Method ^1^	
Uncertain Samples ^2^ (% of Total Samples)			Samples Assigned to a Category ^3^ (% of Total Samples)		Correctly Classified ^4^ (% of Assigned Samples)		Reliable Assignment (%)	
	Mean	SD	Mean	SD	Mean	SD	Mean	SD
**EVOO**	31.0	7.9	69.0	7.9	82.3	15.7	87.5	11.3
**VOO**	26.0	14.4	74.0	14.4	74.6	17.2	80.5	15.5
**LOO**	6.1	6.4	93.9	6.4	80.5	9.7	83.7	9.9
**Total**	23.3	7.4	76.7	7.4	78.9 ^5^	3.83 ^5^	84.0	4.4

^1^ Considering that the analysis of the uncertain samples by the panel test is 100% of correct classification, given that it is the current reference method; ^2^ Samples not assigned to a category by the instrumental screening tool; ^3^ Samples assigned to a category by the instrumental screening tool; ^4^ % of correctly classified samples = (samples correctly classified/all classified samples) × 100; ^5^ Weighted mean and standard deviation.

**Table 3:** External validation classification results for non-EVOO samples (*n* = 36), according to the main perceived defect (MPD) by the panel test: uncertain (%) and correctly classified samples (%).

MPD	Validation Set ^1^	Uncertain Samples (%)	Samples Assigned to a Category	Non-EVOO Samples Assigned to a Category		
			Total (%)	Correct (%) as Non-EVOO ^2^	Correct (%) as VOO ^3^	Correct (%) as LOO ^3^
**RANCID**	1 (*n* = 14)	0 (0/14)	100 (14/14)	71.4 (10/14)	40.0 (4/10)	50.0 (2/4)
	2 (*n* = 13)	46.2 (6/13)	53.8 (7/13)	85.7 (6/7)	100 (2/2)	80.0 (4/5)
	3 (*n* = 12)	16.7 (2/12)	83.3 (10/12)	90.0 (9/10)	80.0 (4/5)	80.0 (4/5)
	4 (*n* = 14)	14.3 (2/14)	85.7 (12/14)	75.0 (9/12)	57.1 (4/7)	60.0 (3/5)
	5 (*n* = 14)	35.7 (5/14)	64.3 (9/14)	88.9 (8/9)	60.0 (3/5)	75.0 (3/4)
	6 (*n* = 12)	33.3 (4/12)	66.7 (8/12)	100 (8/8)	75.0 (3/4)	100 (4/4)
	7 (*n* = 10)	50.0 (5/10)	50.0 (5/10)	100 (5/5)	100 (2/2)	100 (3/3)
**Weighted mean**		**26.9**	**73.0**	**84.6**	**62.9**	**76.7**
**FUSTY-MUDDY**	1 (*n* = 15)	0 (0/15)	100 (15/15)	93.3 (14/15)	77.8 (7/9)	83.3 (5/6)
	2 (*n* = 19)	21.1 (4/19)	78.9 (15/19)	100 (15/15)	100 (12/12)	66.7 (2/3)
	3 (*n* = 17)	35.3 (6/17)	64.7 (11/17)	100 (11/11)	83.3 (5/6)	100 (5/5)
	4 (*n* = 20)	20.0 (4/20)	80.0 (16/20)	87.5 (14/16)	62.5 (5/8)	87.5 (7/8)
	5 (*n* = 15)	20.0 (3/15)	80.0 (12/15)	91.7 (11/12)	66.7 (4/6)	83.3 (5/6)
	6 (*n* = 19)	10.5 (2/19)	89.5 (17/19)	94.1 (16/17)	92.3 (12/13)	75.0 (3/4)
	7 (*n* = 20)	5.0 (1/20)	95.0 (19/20)	90.0 (18/20)	85.7 (12/14)	100 (5/5)
**Weighted Mean**		**16.0**	**84.0**	**93.4**	**83.8**	**86.5**
**MUSTY-HUMID-EARTHY**	1 (*n* = 7)	0 (0/7)	100 (7/7)	85.7 (6/7)	33.3 (1/3)	75.00 (3/4)
	2 (*n* = 4)	0 (0/4)	100 (4/4)	100 (4/4)	100 (1/1)	66.7 (2/3)
	3 (*n* = 7)	28.6 (2/7)	71.4 (5/7)	100 (5/5)	0 (0/2)	100 (3/3)
	4 (*n* = 2)	0 (0/2)	10 (2/2)	100 (2/2)	100 (1/1)	100 (1/1)
	5 (*n* = 7)	14.3 (1/7)	85.7 (6/7)	100 (6/6)	50 (1/2)	75.0 (3/4)
	6 (*n* = 5)	20.0 (1/5)	80.0 (4/5)	100 (4/4)	(0/0)	75.0 (3/4)
	7 (*n* = 6)	33.3 (2/6)	66.7 (4/6)	100 (4/4)	(0/0)	100 (4/4)
**Weighted Mean**		**15.8**	**84.2**	**97**	**44.4**	**82.6**

^1^ All validation sets included 36 non-EVOO samples (22 VOO and 14 LOO). However, the number of samples with rancid (*n* = 10–14), fusty-muddy (*n* = 15–20) or musty-humid-earthy (*n* = 2–7) as main defect differed between sets as a result of random selection according to the commercial category; ^2^ Classification of non-EVOO samples as non-EVOO by the first PLS-DA model (EVOO vs. non-EVOO); ^3^ Classification of non-EVOO samples by the second PLS-DA model (LOO vs. VOO).

## Supplementary Materials

The following are available online at https://www.mdpi.com/2304-8158/9/10/1509/s1. Figure S1: Inter-day (a) and intra-day (b) repeatability plot for the in-house validation of the analytical outcomes. Table S1: Classification results for every virgin olive oil sample in the seven random sets: commercial category assigned by the panel tests, predicted value (PV) and predicted category (PC) for the cross-validation (CV) of each binary model (EVOO/non-EVOO and VOO/LOO) and for the external validation of the whole classification strategy. Table S2: Comparison between alignment approaches: (a) Global alignment and (b) Alignment “per batch” through PCA and PLS-DA outcomes.
